# Isolation and Genetic Characterization of *Streptococcus iniae* Virulence Factors in Adriatic Sturgeon (*Acipenser naccarii*)

**DOI:** 10.3390/microorganisms10050883

**Published:** 2022-04-22

**Authors:** Silvia Colussi, Paolo Pastorino, Davide Mugetti, Elisabetta Antuofermo, Simona Sciuto, Giuseppe Esposito, Marta Polinas, Mattia Tomasoni, Giovanni Pietro Burrai, José Francisco Fernández-Garayzábal, Pier Luigi Acutis, Claudio Pedron, Marino Prearo

**Affiliations:** 1 The Veterinary Medical Research Institute for Piemonte, Liguria and Valle d’Aosta, 10154 Turin, Italy; silvia.colussi@izsto.it (S.C.); simona.sciuto@izsto.it (S.S.); giuseppe.esposito@izsto.it (G.E.); mattia.tomasoni@izsto.it (M.T.); pierluigi.acutis@izsto.it (P.L.A.); marino.prearo@izsto.it (M.P.); 2 Department of Veterinary Medicine, University of Sassari, 07100 Sassari, Italy; eantuofermo@uniss.it (E.A.); mpolinas@uniss.it (M.P.); gburrai@uniss.it (G.P.B.); 3 Centro de Vigilancia Sanitaria Veterinaria (VISAVET), Universidad Complutense de Madrid, 28040 Madrid, Spain; garayzab@ucm.es; 4 Independent Researcher, DVM, 20090 Settala, Italy; claudio.pedron@alice.it

**Keywords:** bacterial diseases, streptococcosis, *Acipenseridae*, virulence factors

## Abstract

The first case of infection of *Streptococcus iniae* in Adriatic sturgeon (*Acipenser naccarii*) was recently reported in a raceway system located in Northern Italy. A second episode of infection in sturgeons with absence of mortality and evident clinical signs, was registered in November 2020 in the same farm and is reported in this study. Histopathological changes observed in infected organs are described. The strains isolated in the two episodes were compared using molecular analysis based on PCR, phylogeny and virulence factors analysis. Not all the major virulence factors were detected for the two strains; in particular the strains 78697, isolated in November, lacks *cpsD*, compared to the strains 64844, isolated in September. Moreover, genetic variations were reported for *lctO* and *pmg* genes. These findings let us hypothesize a different virulence of the strains in accordance with clinical findings related to the sturgeons.

## 1. Introduction

*Streptococcus iniae* has been reported as one of the most important pathogenic bacteria in aquaculture resulting in severe economic losses of hundreds of millions of dollars annually [1]. *S. iniae* is the main causative agent of streptococcosis in wild and farmed fish worldwide, affecting at least 27 different species, including rainbow trout (*Oncorhynchus mykiss*) [2,3,4], Nile tilapia (*Oreochromis niloticus*) [5,6] and its hybrids [7], Siberian sturgeon (*Acipenser baerii*) [8,9], channel catfish (*Ictalurus punctatus*) [10], barramundi (*Lates calcarifer*) [11,12], red drum (*Sciaenops ocellatus*) [13] and Japanese flounder (*Paralichthys olivaceus*) [14,15]. Moreover, *S. iniae* represents a zoonotic risk, usually associated with the handling and preparation of infected fish for consumption [16].

Even if not all the pathogenetic pathway of *S. iniae* is well known, a crucial step is considered the interaction between *S. iniae* and macrophages. Zlotkin and co-authors postulated the “Trojan horse” theory, according to which migrating monocytes are exploited to invade the central nervous system [17]. Moreover, induction of apoptosis in macrophages was also suggested to explain the avoidance of immune system priming. Virulence factors described in experimental infections of white striped bass (*Morone chrysops* × *M. saxatilis*) contribute to *S. iniae* pathogenicity [16]. In particular, seven virulence factors seems to be involved: M-like protein (*simA*) and C5a peptidase (*scpI*) contributing to cellular adhesion and invasion and providing resistance to phagocytic killing [18]; posphoglucomutase (*pgm*), a crucial factor for virulence, involved in cell wall morphology, surface capsule expression and resistance to cationic antimicrobial peptides [19]; the sag operon (*sagA*) involved in the formation of the cytolysin SLS, homologous to streptolysin S of *S. pyogenes*, *sagA* mutant presenting a reduction in their virulence [20]; polysaccharide deacetylase (*Pdi*) found to promote bacterial resistance to lysozyme killing and the ability to adhere to and invade epithelial cells [21]; capsular polysaccharide (*cpsD*) playing a crucial role in the formation of streptococcal species capsule determining virulence and leading to fish kills [20,22]; CAMP factor-like (*cfi*) known to bind immunoglobulin by the Fc region and therefore contribute to virulence [16]. An increased understanding of the properties of the effect of these factors on the success of infection will contribute to use the appropriate approach to control *S. iniae* infection in Adriatic sturgeon, avoiding heavy economic losses for Italian sturgeon farming and caviar production and risks for the wild populations in restocking procedures.

As previously mentioned, *S. iniae* has been recognized as a cause of high mortality in farmed rainbow trout and Nile tilapia [23]. Furthermore, several reports of infections by this pathogen on different sturgeon species are known, including Siberian sturgeon [8], Chinese sturgeon (*A. sinensis*) [24], white sturgeon (*A. transmontanus*) [25,26], *Huso dauricus* × *A. schrenckii* hybrids [27] and recently Adriatic sturgeon (*A. naccarii*) [28]. According to our knowledge, the presence of *S. iniae* in Adriatic sturgeon has been reported for the first time in 2020: in fact, until 2018 it was not related to *Acipenseridae* mortality events in Italy, as monitored by Santi and co-authors [29].

This paper is focused on the description of a second infective episode following the case of mortality described by Mugetti et al. [28], considering histological aspects, phenotypic and molecular identification of the strains and comparison among the isolates using molecular biology as tools for phylogeny and virulence factors analysis. Considering that *S. iniae* diagnosis is usually difficult using conventional biochemical tests, molecular diagnostic tools were selected in this study to reach an accurate identification of the pathogen. The study of the 16S rRNA gene [30] was carried out and amplicons were used also for the phylogenetic analysis; moreover, target specific PCR based on the lactate oxidase (*lctO*) gene was applied for a specific identification [31].

## 2. Materials and Methods

### 2.1. Fish Sampling and Differential Diagnosis

The first episode of mortality in Adriatic sturgeon was registered during the month of September 2020 in a raceway system from a farm that breeds several sturgeon species. A few months later, in November 2020, an alteration of the health state of sturgeon, in absence of mortality, was reported, mainly characterized by starvation and arching of the back.

Four fish were analyzed by the Veterinary Medical Research Institute for Piemonte, Liguria and Valle d’Aosta (IZSPLV). Total length (Lt; cm) and total weight (W; g) were recorded for each specimen. Externally, two fish (78697.3 and 78697.4) were apparently healthy, while specimen 78697.1 was characterized by starvation and skeletal deformities; spinal change was also reported for specimen 78697.2.

The present work does not include any experimental infections trial with Adriatic sturgeon, just sturgeons that were exclusively used for the microbiological identification of the etiological agent. In order to ensure the welfare and ameliorate suffering of surgeons during transportation to the laboratory and euthanasia, fish were handled according to guidelines of relevant international organisms such as OIE (http://www.oie.int/doc/ged/D7821.PDF) (accessed on 21 February 2022) and AVMA (https://www.avma.org/KB/Policies/Documents/euthanasia.pdf (accessed on 21 February 2022)) and they were further necropsied under aseptic conditions. The animals were suppressed by a lethal dose of tricaine methanesulphonate anesthetic (MS-222; Sigma-Aldrich, St. Louis, MO, USA) and necropsied, and samples were collected for histopathological, microbiological, parasitological and virological analyses. Necropsy and parasitological examination were conducted as described by Mugetti et al. [28]. The tissues (gill, liver, spleen and intestine) for histopathology were processed by standard paraffin wax techniques and stained with haematoxylin-eosin (H.E.). Gill lamellae were taken for the detection of Acipenser Iridovirus European (AcIV-E) by real-time PCR with the protocol of Bigarré et al. [32]. Briefly, a 30 mg sample was used for total DNA extraction using ExtractMe kit (Blirt, Gdańsk, Poland) following the instructions for fresh solid tissues. The extracts were then tested by a real-time PCR targeting the major capsid protein of AcIV-E using primers oPVP346 and oPVP347 and probe tqPVP20; the reactions were carried out in a total volume of 25 μL using a BioRad CFX96 Touch Real-Time PCR Detection System (Bio-Rad, Hercules, CA, USA).

### 2.2. Bacterial Culture and Phenotypic Identification

Bacteriological analysis of the kidney, eyes and brain was carried out as described by Santi et al. [29] for the monitoring previously conducted on sturgeon farms. All isolated colonies were cloned on Columbia blood agar and sent for classification by biochemical tests and Maldi-Tof (Bruker Daltonics Inc., Billerica, MA, USA). Biochemical tests on micro-method were performed using API^®^ 20 Strep and API^®^ Rapid ID 32 Strep galleries (bioMérieux, Marcy-l’Étoile, France) following the manufacturer’s instructions.

### 2.3. Kirby–Bauer Susceptibility Test

The strains susceptibility to several antibiotics was conducted by the Kirby–Bauer agar diffusion method. The antibiograms were prepared on Müller–Hinton Agar with 5% Sheep Blood plates and incubated for 48 h at 22 ± 2 °C, after which the inhibition zone was manually measured. The following antibiotics were tested (standard laboratory panel): ampicillin (10 μg), amoxicillin (25 μg), chloramphenicol (30 μg), erythromycin (15 μg), enrofloxacin (5 μg), florfenicol (30 μg), furazolidone (100 μg), gentamicin (10 μg), lincomycin (2 μg), oxitetracycline (30 μg), tetracycline (30 μg), thiamphenicol (30 μg), trimethoprim + sulfamethoxazole (1.25 μg + 23.75 μg), kanamycin (30 μg), penicillin (10 IU), streptomycin (10 μg) and spiramycin (100 μg). The reference strain *S. iniae* DSM 20576 was used as internal quality control. Each antibiotic inhibition zone was translated into a category (susceptible, intermediate and resistant) according to the Clinical and Laboratory Standards Institute (CLSI) guidelines for bacteria isolated from aquatic animals [33,34].

### 2.4. Histopathology

Tissue samples from the spleen, liver, kidney and intestine of four adult sturgeons were collected and immediately fixed in 10% buffered formalin, dehydrated with increasing alcohol concentrations and xylene in an automatic tissue processor and paraffin-embedded. Sections of three μm thickness were stained with hematoxylin and eosin (H.E.) in an automatic multistainer (ST5020, Leica Biosystems, Wetzlar, Germany) and with Gram staining (Bio-Optica, Milano, Italy). Slides were then evaluated at light microscopy (Nikon Eclipse 80i) (Nikon, Tokyo, Japan) equipped with a digital camera.

Brain samples were included in the histological analysis, moreover, due to the poor state of brain tissue conservation (i.e., advanced autolysis), no histological description was performed.

### 2.5. Molecular Identification, Pathogenicity and Phylogenesis

DNA extraction of pure colonies isolated from the brains was performed using the boiling and freeze–thawing protocol as described by Pastorino and co-authors [35]. The bacterial 16S rRNA and lactate oxidase (*lctO*) genes were amplified according to the protocols described by Jensen and Mata, respectively [30,31]. Amplicons were run on 2% Gelgreen (Biotium, Fremont, CA, USA) stained agarose gel and then visualized under UV exposure. A 50–2000 kb ladder (Amplisize Molecular Ruler, Bio-Rad, Hercules, CA, USA) was used as a molecular marker. The amplicons were purified with an ExtractMe DNA Clean-up and Gel-out kit (Blirt, Gdańsk, Poland) according to the manufacturer’s instructions. The purified PCR products were bidirectionally sequenced using Big Dye 1.1 and 3.1 (Applied Biosystems, Waltham, MA, USA) chemistries and using the same primers of PCR amplification. Cycle sequencing products were purified using Dye Ex 2.0 spin kit (Qiagen, Hilden, Germany) and sequenced in an ABI3130xl Genetic analyzer (Applied Biosystems, Waltham, MA, USA).

Contig assembly of forward and reverse DNA sequences were performed using Lasergene Software package (DNASTAR). The 16S rRNA gene sequences were compared with nucleotide sequences in the GenBank ^®^ database using the Basic Local Alignment Search Tool (BLAST) search algorithm. Moreover, a neighbor-joining analysis was performed [36] on 16S rRNA sequences of the isolated strains and other *S. iniae* sequences belonging to different fish species and amphibians using Molecular Evolutionary Genetics Analysis software (MEGA; Ver. 7.0), with evolutionary distances ascertained via the maximum composite likelihood method [37]. A bootstrap test of 1000 replicates was performed. *S. dysgalactiae* and *S. agalactiae* sequences were considered as an output group in analogy to what has been reported by Liu et al. [38]. Isolated strains were also investigated for the presence of seven virulence genes: C5a peptidase (*scpI*); M-like protein A (*simA*); capsular polysaccharide (*cpsD*); Sag operon (*sagA*); polysaccharide deacetylase (*pdi*); phosphoglucomutase (*pgm*); and CAMP factor-like (*cfi*) by PCR [6]. PCR products were amplified and sequenced, when present, as described above.

## 3. Results

### 3.1. Biometrical Features of Sturgeons

Values of total length and weight of sturgeons are reported in Table 1.

### 3.2. Differential Diagnosis

Parasitological tests did not reveal the presence of macro and microscopic parasites. Similarly, the tests for AcIV-E detection gave negative results.

### 3.3. Bacterial Culture, Phenotypic and Proteomic Identification

Small, whitish-gray colonies, surrounded by a β-haemolysis area, grew on Blood Agar plates. Microscopy analysis after Gram staining allowed to highlight Gram positive cocci. Regarding the biochemical tests, isolated strains appeared acid producers from maltose, mannitol, ribose, saccharose, starch and trehalose, while they were uncapable to produce acid from d-arabitol, inulin, lactose, l-arabinose, melibiose, raffinose, sorbitol and tagatose. The strains produced pyrrolidonyl arylamidase, alkaline phosphatase, leucine arylamidase, and arginine dihydrolase; α- and β-Galactosidases and β-glucuronidase were not produced. Furthermore, esculin was hydrolyzed, but hippurate not. All colonies isolated and tested at Maldi-Tof were accurately identified as *S. iniae*.

### 3.4. Antimicrobial Resistance

Following the Kirby–Bauer test, strains resulted susceptible to ampicillin, amoxicillin, chloramphenicol, erythromycin, enrofloxacin, florfenicol, furazolidone, gentamicin, lincomycin, thiamphenicol, trimethoprim plus sulfamethoxazole, penicillin and spiramycin, but intermediate to kanamycin and resistant to streptomycin, oxitetracycline and tetracycline.

### 3.5. Histopathology

**Figure 1:**
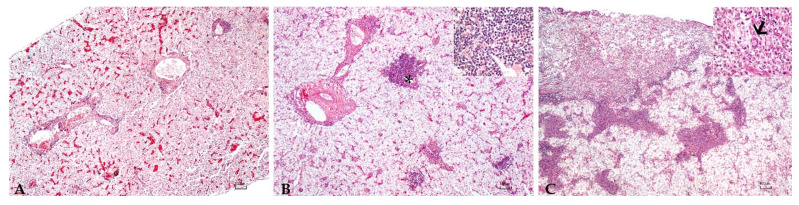
**Liver**: Mild (**A**), moderate (**B**), and severe (**C**) granulomatous chronic hepatitis cantered around the bile ducts and arterial vessels (asterisk, (**B**)). Granulomas consisted of a high number of macrophages, a moderate number of lymphocytes and plasma cells, scattered eosinophilic granular cells (inset (**B**)), and occasionally 80–100 µm, round, multinucleated cells with round peripherally-located nuclei with dispersed chromatin and granular eosinophilic cytoplasm (Langhans-type cells) (arrow, inset (**C**)). H.E. Bar (**A**–**C**) = 100 µm, inset 10 µm.

**Figure 2:**
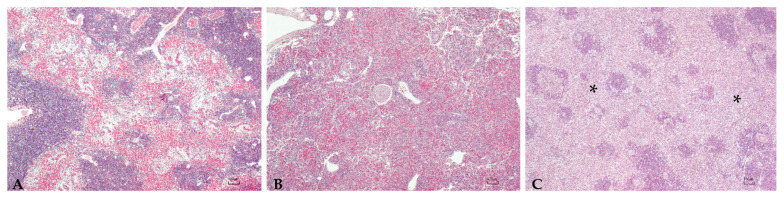
**Spleen:** Mild (**A**), moderate (**B**), and severe (**C**) granulomatous chronic splenitis mainly involving the red pulp (asterisk (**C**)), constituted of a high number of activated macrophages. H.E. Bar (**A**–**C**) = 100 µm.

**Figure 3:**
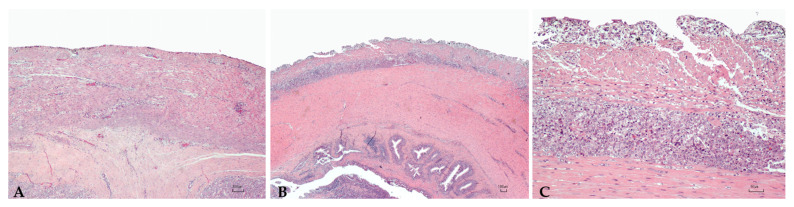
Intestine. Mild (**A**) and severe (**B**,**C**) granulomatous chronic enteritis. (**C**): High magnification, Table 2. (**B**) showing a high number of activated macrophages located in the serosa and the outer muscular layer. H.E. Bar (**A**–**C**) = 100 µm.

Sturgeons showed mild to severe granulomatous chronic inflammatory reactions ranging from mild to severe degrees in all examined organs. In particular, the hepatic parenchyma was expanded by multifocal to coalescing, multinodular, variable size granulomas mainly located near vessels and bile ducts and extended to the serosal surface in the most severe cases (Figure 1). Mild and diffuse congestion of blood vessels was observed in a single case with mild inflammation. The spleen was diffusely expanded in all sturgeons except in one specimen, by a mild to severe chronic granulomatous inflammation associated with edema and vascular congestion (Figure 2). In two out of four sturgeons, the serosa and the outer muscular layer of the small intestine were characterized by a diffuse granulomatous inflammation, whereas the mucosa and submucosa were unaffected (Figure 3). Results are summarized in Table 2.

### 3.6. Molecular Identification, Pathogenicity and Phylogenesis

The isolates 78697 showed a 1500 bp band on agarose gel for 16s RNA gene and were identified as *S. iniae* (BLASTn nucleotide sequence identity value of 99,78, e-value: 0.0, compared to *S. iniae* strain 2009001, GenBank^®^ Accession Number MW455461); the sequence obtained was deposited to GenBank^®^ (Accession number OK642580). While in the paper published by Mugetti and co-authors [28] identity values for strains 64844 were 100%. Specific amplicons of 870 bp were obtained using a PCR based on the lctO gene, but a variation among the sequences was reported. The LctO gene of strain 64844 presented a 99.75% similarity with the reference sequence Y07622. Two single nucleotide variations (SNPs) were found at position 3290 (T > C) and 3633 (T > G) causing, respectively, a synonym substitution Val>Val and a substitution Ser>Ala in the aminoacidic sequence. The substitution Ser>Val showed a positive score in the mutation matrix attributable to a common presence in nature, indicating a favorable substitution. The LctO of the strain 78697 showed lesser similarity to the reference sequence (99.53%) due to the presence of three SNPs and a one bp deletion. In this case the same SNPs of the strain 64844 were reported at positions 3290 and 3633, in addition with a variation at position 3689 (T > C), causing a synonymous mutation of the aminoacidic sequence (His > His). Interestingly, the deletion caused a frameshift of the Open Reading Frame causing the appearance of a stop codon before the natural end of the sequence, generating a 389 instead a 403 aminoacidic sequence; the sequence was deposited to GenBank^®^ (Accession number OL753436).

Table 3 summarizes the results obtained for the amplification of the seven virulence factors. Of particular relevance was the absence of cpsD in the strain 78697. Moreover, the differences were reported among the sequences for the pgm gene. An SNP at position 1043 t > g of the pgm gene (reference sequence GenBank^®^ accession number JF795256) was reported for strain 64844. The presence of the variant g was found in the strain 64844 and was associated to a non-conservative aminoacidic substitution from Valine to Glycine at position 348 of the protein. (BLASTn identity value = 99.86%; e-value: 0.0). The sequence has been deposited to GenBank^®^ (Accession number OL753438).

**Figure 4:**
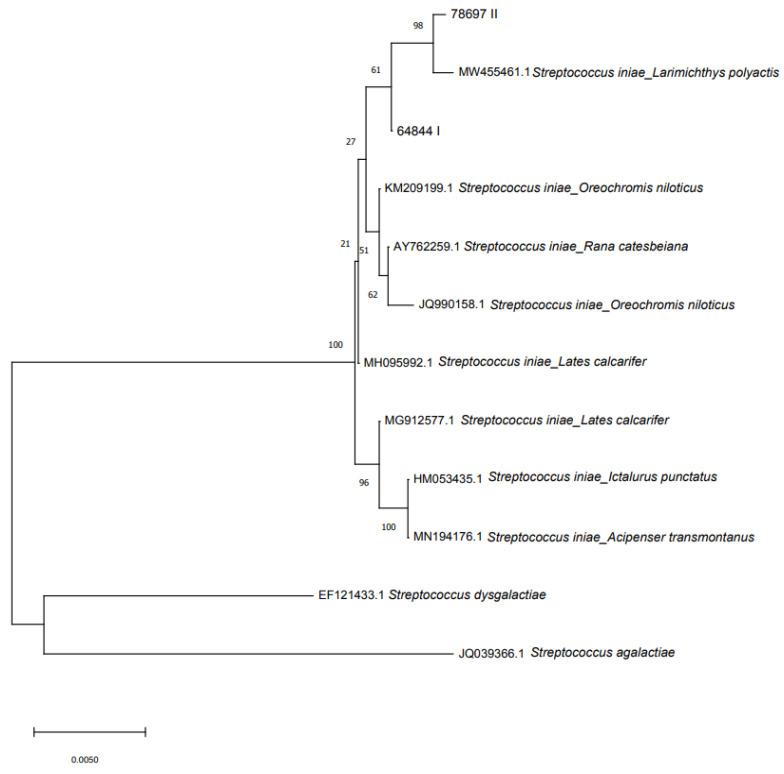
Phylogenetic relationship based on 16S rRNA gene sequences of *S. iniae* isolated from sturgeon with other NCBI reported sequences of *Streptococcus*. The phylogenetic tree was constructed using MEGA 7 and the neighbor-joining method.

Phylogenetic analysis showed intraspecies genotypic variability within the *S. iniae* clade (Figure 4 displays the original tree). Two *S. iniae* clusters were present: the first comprising the two sturgeon isolates and 16S rRNA gene sequences from *S. iniae* isolated from different species (MW455461 *Larimichthys polyactis*; KM209199 *Oreochromis niloticus*; AY762259 *Rana catesbeiana*; JQ990158 *Oreochromis niloticus*; MH095992 *Lates calcarifer*). The second cluster was made by three sequences of *S. iniae* isolated from *L. calcarifer*, *Ictalurus punctatus* and *A. transmontanus*.

## 4. Discussion

The previous paper of Mugetti et al. [28] showed clinical signs consistent with those ready reported regarding *A. baerii* [8] and *A. transmontanus* [25]. In this second episode two sturgeons were externally normal, even if the histopathological analysis reported a mild inflammation of the liver and the spleen; other two samples were instead characterized by starvation and skeletal deformities, as already reported for *A. transmontanus* [26]. Moreover, the multifocal involvement of the liver, spleen, and of the intestine mainly centered around vessels and bile ducts, indicating a systemic granulomatous inflammation, and was reported.

The performed analysis confirmed the presence of *S. iniae*, excluding the presence of other bacteria, parasites and AcIV-E. In our study, Gram-positive streptococci were the only detected pathogens, thus they can be considered to have been responsible for the observed alteration of the health state of examined sturgeons. The strains were identified by biochemical and molecular methods allowing a confident identification of *S. iniae* in both registered episodes. Nevertheless, variations among the strains were reported for *lctO* gene, reported as a genetic marker for the rapid and specific detection and identification from *S. iniae* from different sources [31]. Other authors described a different form of the *lctO*, with an insertion producing an amplicon with a different size (920 bp instead of 870) [39].

The *lctO* gene of the strain 78697 exhibited a one bp deletion causing a frameshift of the open reading frame, inserting a stop codon 14 aminoacids before the normal aminoacidic sequence. Lactate can be utilized as energy source transforming lactate to pyruvate. It could be speculated that modification in *lctO* gene in the strain 78697 could have altered the oxidative function of the enzyme lactate oxidase, affecting its growth rate ability and consequently its propagation capability during infection.

A large panel of antibiotics was tested to look for possible resistance. Fortunately, the isolated strains were sensitive to most of the antibiotics tested, especially β-lactams and macrolides. Based on these results, all the drugs approved in Italy for aquaculture could be used (amoxicillin, florfenicol, trimethoprim plus sulfamethoxazole, and erythromycin used under derogation), except for tetracycline. Despite this, any new Italian isolates of *S. iniae* must be tested for antibiotics sensitivity, given that resistant strains have been already recovered from sturgeons [40].

Not all the major virulence factors were detected for the strains isolated in the two episodes; in particular the strain 78697 lacks *cpsD*, compared to the strain 64844. One of the most effective ways for a bacterium to avoid phagocytosis is by the production of capsular polysaccharide, and strains with CPS are more virulent than their unencapsulated counterparts [22]. The absence of this factor could reduce the ability of the strain 78697 to survive phagocytosis and escape the immune system. Moreover, variations were also reported for the *pmg* gene.

The *Pgm* gene encoding for the enzyme phosphoglucomutase, interconverting glucose-6-phosphate and glucose-1-phosphate, was instead reported in both strains but with an SNP variation between the sequences. Variation in the nucleic and aminoacidic sequence could be related to alterations in cell wall morphology, capsule production, and susceptibility to fish innate immune defense [19]. In this case, the variation 1043G could have contributed to increase the virulence of the strain 64844.

The *ScpI* gene was not found in both strains; this gene was described in the *S. iniae* virulent strain as homologous of the C5a peptidase with a virulence factor capable of inactivating the complement derived neutrophil chemoattractant C5a, already reported for group *Streptococcus pyogenes*. Notably, allelic exchange has shown that this protein by itself is not required for virulence in fish and its role in pathogenesis is likely a minor one [18].

From a phylogenetic point of view, based on the 16S rRNA gene, the two strains were similar and, as expected, were grouped in the same cluster.

The findings reported above, allow us to hypothesize a different virulence of the two strains in accordance with clinical findings related to the sturgeons; in fact, isolate 64844 came from the first episode of mortality registered in September 2020, while strain 78697 was isolated from the brain of an apparently healthy sturgeon.

Even if not fully proven in our case due to the lack of histological examination of the brain, it is possible, according to the “Trojan horse mechanism”, that *S. iniae* enter and multiply within migrating monocytes where the pathogen gains an efficient mechanism for translocation into the central nervous system and, under favorable condition (i.e., stressful events), can spread into different organs, leading to multifocal granulomatous inflammations and clinical signs.

The multinucleated cells are noteworthy. Although seldom reported in the fish inflammatory response of bacterial origin, the specimens herein described were found in association with reactive macrophages. These results suggest that these cells in sturgeons could be part of granulomatous inflammation against bacteria.

Despite differences in the virulence factors that could explain the differences in the pathogenicity between the strains analyzed in the two episodes, the influence of water temperature should be also considered.

High water temperatures have been related with a greater severity of symptoms, as well as higher mortality rates associated with infections by warm-water fish pathogens [41]. The first mortality episode was detected in September, a month in which water temperatures are commonly warm, while the second episode occurred in November when water temperatures were lower. Thus, the differences in the water temperature may have contributed to the lower severity of symptoms in the second infective episode.

Clinical and subclinical infections due to *S. iniae* should be taken into account, considering the high economic losses that Italian caviar production could encounter. Moreover, several native species are bred for restocking programs of natural fish stocks [42] (e.g., *A. naccarii*) and for this reason, subclinical infections have to be considered to be of particular concern due to the possibility of transmission to other species in the natural environment. In this perspective, constant monitoring combined with the characterization of the isolates are of fundamental importance for the control of *S. iniae* in Italy.

## Figures and Tables

**Table 1:** Identification code (ID), total length (cm) and weigh (g) of the four sturgeons.

ID	Total Length (cm)	Weight (g)
78697.1	94.5	4100
78697.2	81.5	2800
78697.3	96.2	3700
78697.4	98.5	3800

**Table 2:** Histopathology.

ID	Liver	Spleen	Gut
78697.1	Severe inflammation	Severe inflammation	Severe inflammation
78697.2	Moderate inflammation	Mild inflammation/severe congestion	Mild inflammation
78697.3	Mild inflammation/severe congestion	Moderate inflammation	No lesion
78697.4	Moderate inflammation	No lesion	No lesion

**Table 3:** Results obtained for the virulence factors amplification and their specific length.

	*scpI* (822 bp)	*simA* (994 bp)	*pdi* (381 bp)	*sagA* (190 bp)	*cpsD* (534 bp)	*pgm* (713 bp)	*cfi* (328 bp)
64844	−	+	+	+	+	+	+
78697	−	+	+	+	−	+	+

## Data Availability

Sequences were deposited to GenBank ^®^ (https://www.ncbi.nlm.nih.gov/genbank/; accessed on 20 March 2022) a publicly available sequences database with the following accessions numbers: OK642580 for 16S RNA gene; OL753436 for *lctO* gene; OL753438 for *pgm* gene).
